# The Role of Re-Entrant Microstructures in Modulating Droplet Evaporation Modes

**DOI:** 10.3390/mi15121507

**Published:** 2024-12-18

**Authors:** Hoang Huy Vu, Nam-Trung Nguyen, Navid Kashaninejad

**Affiliations:** Queensland Micro- and Nanotechnology Centre, Griffith University, Nathan Campus, 170 Kessels Road, Brisbane, QLD 4111, Australia; hoang.vu@griffith.edu.au

**Keywords:** re-entrant structures, silicon carbide, silicon oxide, wettability, evaporation

## Abstract

The evaporation dynamics of sessile droplets on re-entrant microstructures are critical for applications in microfluidics, thermal management, and self-cleaning surfaces. Re-entrant structures, such as mushroom-like shapes with overhanging features, trap air beneath droplets to enhance non-wettability. The present study examines the evaporation of a water droplet on silicon carbide (SiC) and silicon dioxide (SiO_2_) re-entrant structures, focusing on the effects of material composition and solid area fraction on volume reduction, contact angle, and evaporation modes. Using surface free energy (SFE) as an indicator of wettability, we find that the low SFE of SiC promotes quick depinning and contact line retraction, resulting in shorter CCL phases across different structures. For instance, the CCL phase accounts for 55–59% of the evaporation time on SiC surfaces, while on SiO_2_ it extends to 51–68%, reflecting a 7–23% increase in duration due to stronger pinning effects. Additionally, narrower pillar gaps, which increase the solid area fraction, further stabilize droplets by extending both CCL and constant contact angle (CCA) phases, while wider gaps enable faster depinning and evaporation. These findings illustrate how hydrophobicity (via SFE) and structural geometry (via solid area fraction) influence microscale interactions, offering insights for designing surfaces with optimized liquid management properties.

## 1. Introduction

Droplet evaporation on solid surfaces has been widely studied due to its relevance in applications like inkjet printing, spray coating, cooling systems, and microfluidics [1,2,3,4,5,6,7,8,9]. In scientific and industrial contexts, a thorough understanding of sessile droplet evaporation is crucial for optimizing processes such as nanopatterning, cell culture, and pesticide delivery [10,11,12,13]. The evaporation behavior of a droplet is largely influenced by its interactions with the surface, including parameters like surface roughness, wettability, and ambient conditions, which collectively govern evaporation rate, contact angle, and contact line dynamics [14]. Surface wettability, determined by chemical composition and texture of the surface, is a key factor influencing droplet behavior during evaporation [15,16].

Surfaces engineered with micro- or nanostructures have been used to manipulate wettability, allowing for precise control over liquid behavior [17]. Superhydrophobic surfaces, in particular, have garnered attention for their ability to maintain high contact angles and minimal surface adhesion, which can either facilitate longer droplet lifetimes or increase evaporation rates depending on environmental conditions and heat transfer dynamics. Among superhydrophobic designs, micropillar arrays, hierarchical structures, and re-entrant microstructures each exhibit unique characteristics [18]. Within the realm of superhydrophobic surfaces, re-entrant microstructures stand out due to their overhanging geometries, which trap air beneath droplets and create a composite interface, known as the Cassie–Baxter state [18,19,20]. In this state, droplets exhibit high contact angles and reduced solid–liquid contact, maintaining a non-wetting behavior during the early stages of evaporation. Compared to conventional micropillar arrays, which can experience transitions to the Wenzel state under certain conditions, re-entrant structures provide greater stability against liquid infiltration. Similarly, while hierarchical surfaces combine micro- and nanoscale roughness for enhanced hydrophobicity, their performance can vary depending on structural robustness and environmental factors [18,21,22,23].

Re-entrant structures, characterized by overhanging or mushroom-like geometries, are designed to trap air beneath droplets, enhancing non-wettability and maintaining a Cassie–Baxter state [18]. Despite extensive research on evaporation dynamics on smooth and conventionally patterned surfaces, the behavior of droplets on re-entrant microstructures remains underexplored. The geometric complexity of these structures adds a new layer of intricacy to the evaporation process. As evaporation progresses, the transition from the Cassie–Baxter state to the Wenzel state, where the liquid fully wets the surface, can significantly affect both the contact line and contact angle, potentially altering the evaporation mode and increasing the evaporation rate [24]. The role of pinning forces in stabilizing or destabilizing the triple line during these transitions has been highlighted by Zinigrad (2011), underscoring the importance of surface properties in modulating evaporation dynamics [16].

Previous studies have largely focused on understanding droplet evaporation on microstructured surfaces, where the process typically follows three modes: constant contact line (CCL), constant contact angle (CCA), and a mixed mode [15,25,26]. McHale et al. examined water droplet evaporation on micropillar surfaces, identifying a mode where droplets transition from the Cassie–Baxter state (sitting on top of pillars) to the Wenzel state (penetrating air pockets) [27]. In the Cassie–Baxter state, droplets initially evaporated in CCL mode, but CCA dominated after shifting to the Wenzel state. Gurrala et al. investigated water droplet evaporation on microstructured surfaces with varying wettability, showing that evaporation dynamics differ significantly from smooth surfaces, primarily following the CCL mode. The findings emphasize that biphilic and hydrophobic surfaces exhibit similar evaporation rates for larger droplets, but as droplet size decreases to the micropillar scale, the structured surface begins to notably impact evaporation [28]. Xu et al. studied the evaporation kinetics of sessile water droplets on superhydrophobic surfaces with micropillars, observing two main modes: CCL and CCA [29]. The study found that a lower solid fraction on the surface shortened the CCL phase and prolonged the CCA phase, affecting overall evaporation due to reduced droplet boundary pinning. Another study by Kashaninejad et al. examines how micropatterned surfaces with varying eccentricities influence the evaporation behavior of sessile water droplets [15]. The researchers fabricated microhole arrays with different eccentricity values on polydimethylsiloxane (PDMS) surfaces and observed that surfaces with non-zero eccentricity exhibited primarily a CCL mode, while surfaces with zero eccentricity showed more mixed-mode evaporation. Contact angle hysteresis (CAH) was found to increase with eccentricity, correlating with the observed evaporation behavior. However, in re-entrant microstructures with overhang structures, these evaporation modes can be modulated by the presence of air pockets and the surface’s ability to maintain a superhydrophobic state. Additionally, research has shown that micropatterned surfaces with engineered wettability and overhang features can affect the pinning and depinning of the droplet’s contact line, leading to transitions between different evaporation modes [18,19,22,30].

The observations from previous studies highlight a knowledge gap regarding the specific influence of re-entrant microstructures on evaporation dynamics. This study aims to bridge this by systematically investigating how re-entrant microstructures influence key evaporation parameters, including droplet volume reduction, contact angle behavior, and contact line dynamics. By focusing on the role of material composition and microstructure geometry in modulating these parameters, we seek to provide insights that will advance the design of surfaces for applications like anti-icing, cooling, and microfluidic devices, where controlled droplet behavior is essential. Through experimental observations and theoretical analysis, this work aims to provide new insights into how re-entrant microstructures can be optimized for improved evaporation control and surface performance across a wide range of applications.

## 2. Materials and Methods

### 2.1. Fabrication of the Re-Entrant Structure

The process of creating micropillars with re-entrant structures began with the preparation of a clean, thermally oxidized SiO_2_ wafer. A layer of SiO_2_ was grown on the wafer, followed by the application of a photoresist layer through spin coating. The wafer was then exposed to ultraviolet (UV) light and developed to create a patterned photoresist. Anisotropic reactive ion etching (RIE) was used to etch the SiO_2_ and form vertical pillars. After stripping the photoresist, a second anisotropic RIE was performed to etch both SiO_2_ and Si layers. To achieve the re-entrant structures, isotropic etching with SF_6_ at a high pressure was applied, creating a cap on top of the pillars made of SiO_2_. Finally, a layer of C_4_F_8_ was coated on the resulting structure. The fabrication process for SiC re-entrant microstructures followed the same steps as previously described, with the primary difference being the use of SiC instead of SiO_2_. This method was detailed in our previous paper [19]. The detailed process is explained in the Supplementary Materials Section.

### 2.2. Evaporation Test

In this study, we observed droplet evaporation dynamics using an optical tensiometer (Theta Flex, Biolin Scientific, Espoo, Finland). Prior to each experiment, the instrument was calibrated optically at a fixed magnification with a 4 mm spherical calibration ball supplied by the manufacturer. For each trial, a small droplet of deionized (DI) water with an initial volume of around 5–7.5 µL was carefully placed on the surface. Room conditions, including temperature and humidity, were closely monitored using a Humidity–Temperature Logger (Lascar Electronics Co., Salisbury, UK), with an average temperature of 26 °C and relative humidity of 36%. A climate control system maintained the laboratory environment, with continuous temperature monitoring provided by a calibrated digital thermometer. Samples were allowed to stabilize at the set temperature for at least 30 min before measurements to ensure thermal equilibrium. This controlled environment minimized thermal effects on both the liquids and re-entrant structures, enhancing the reliability and accuracy of the experimental data. The tensiometer recorded images every 9 s, enabling real-time monitoring of the droplet’s evaporation over a 2 h period, though this timeframe exceeded the actual evaporation duration for all samples. During the experiment, the contact angle (CA), contact baseline length, and droplet volume were measured.

Each experiment was repeated at least three times to ensure reliability. The average values for evaporation time, contact angle, and volume reduction were calculated, with error margins expressed as mean ± standard deviation. This statistical approach confirms consistency and accounts for variability across trials.

The droplet base radius ($r_{b}$) refers to the radius of the droplet’s contact area with the surface, providing insights into the spread of the droplet as it evaporates. The droplet lifetime ($t_{f}$) is the total time taken for the droplet to completely evaporate, measured from the initial state until the droplet vanishes. To standardize measurements, we define the normalized contact radius $r_{b}^{*}$ ($r_{b}^{*}$ = $r_{b,t}/r_{b,0}$), which expresses the droplet base radius at any time *t*, $r_{b,t}$, as a ratio of its initial radius, $r_{b,0}$, allowing us to track radius changes over time in a comparable way.

Additionally, we use normalized time $t^{*}$ ($t^{*}=t/t_{f}$) which represents the elapsed time as a fraction of the droplet’s total lifetime, enabling the consistent analysis of the evaporation process across droplets with varying lifetimes. These parameters collectively allow for a detailed and standardized examination of the droplet’s geometrical evolution and evaporation behavior. These parameters were analyzed using the recorded video footage until the droplet had fully evaporated.

An uncertainty analysis was conducted to ensure that the observed differences in CCL and CCA durations between SiO_2_ and SiC were not due to experimental variability. By comparing the measured differences to the calculated error margins, we confirmed that the variations fall outside the range of experimental error. This approach validates that the observed changes in evaporation modes are statistically significant, reflecting true differences in pinning effects between the materials.

**Figure 1:**
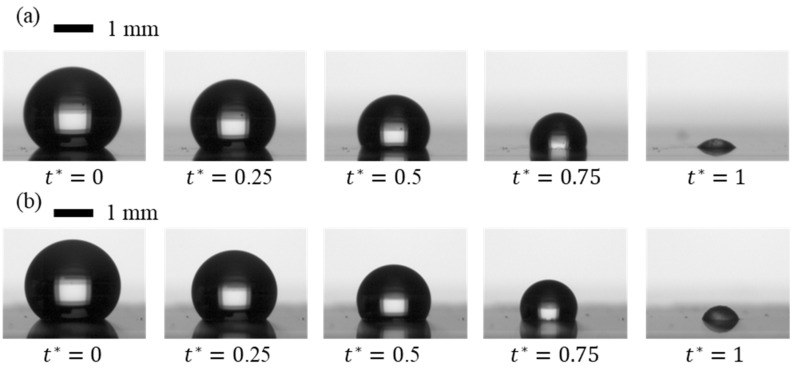
Image sequences of evaporating sessile DI water droplets on re-entrant structures with circular caps: (**a**) SiC cap with 5 µm gap spacing; and (**b**) SiO_2_ cap with 5 µm gap spacing. Scale bar: 1 mm.

Figure 1 presents an image sequence illustrating the evaporation of DI water droplets on SiC and SiO_2_ re-entrant structures with circular caps. Comparing the droplet profiles in Figure 1a,b qualitatively suggest that the material of the circular caps (SiC versus SiO_2_) plays a role in influencing evaporation behavior. Detailed quantitative analyses will be provided in the next section.

### 2.3. Characterization of Micropillar Surfaces

**Figure 2:**
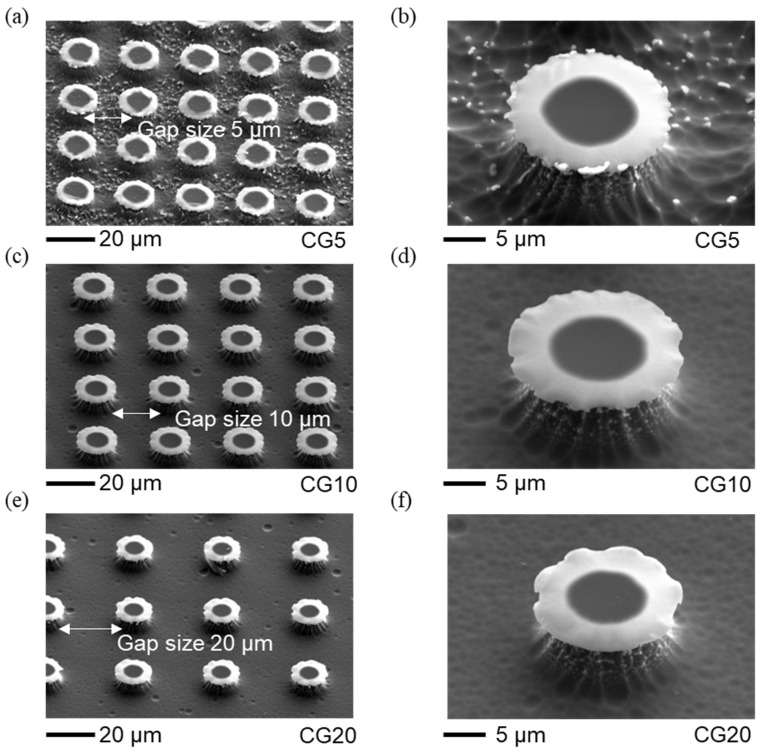
Representative SEM images of the re-entrant structures with SiO_2_ caps of circular design at varying gap distances. The average height of the pillars is approximately 9 µm. (**a**) Top view SEM image of re-entrant structures with circular caps and a gap of 5 µm. Scale bar: 20 µm. (**b**) Close-up SEM image of re-entrant structures with circular caps and a gap of 5 µm. Scale bar: 5 µm. (**c**) Top view SEM image of re-entrant structures with circular caps and a gap of 10 µm. Scale bar: 20 µm. (**d**) Close-up SEM image of re-entrant structures with circular caps and a gap of 10 µm. Scale bar: 5 µm. (**e**) Top view SEM image of re-entrant structures with circular caps and a gap of 20 µm. Scale bar: 20 µm. (**f**) Close-up SEM image of re-entrant structures with circular caps and a gap of 20 µm. Scale bar: 5 µm.

**Figure 3:**
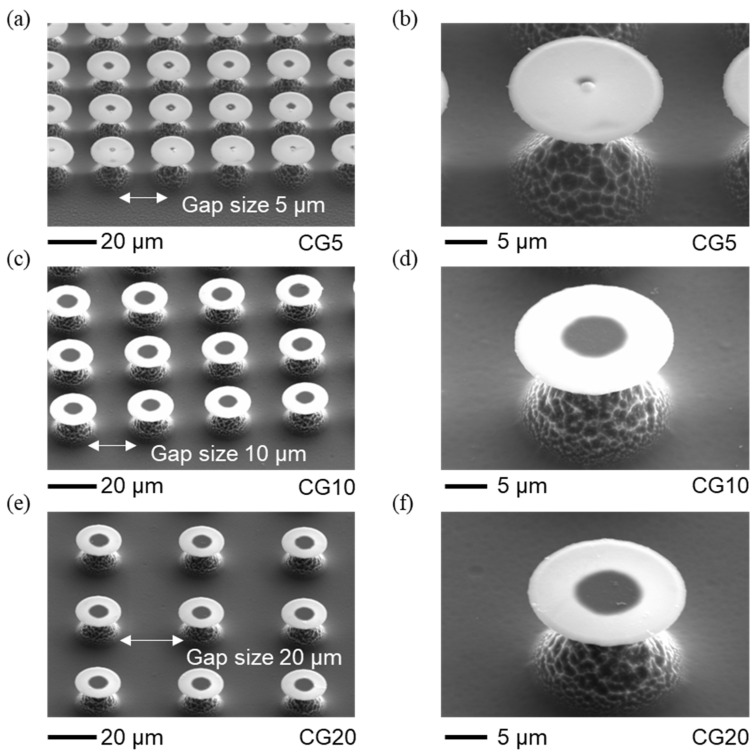
Representative SEM images of the re-entrant structures with SiC caps of circular design at varying gap distances. The average height of the pillars is approximately 12 µm. (**a**) Top view SEM image of re-entrant structures with circular caps and a gap of 5 µm. Scale bar: 20 µm. (**b**) Close-up SEM image of re-entrant structures with circular caps and a gap of 5 µm. Scale bar: 5 µm. (**c**) Top view SEM image of re-entrant structures with circular caps and a gap of 10 µm. Scale bar: 20 µm. (**d**) Close-up SEM image of re-entrant structures with circular caps and a gap of 10 µm. Scale bar: 5 µm. (**e**) Top view SEM image of re-entrant structures with circular caps and a gap of 20 µm. Scale bar: 20 µm. (**f**) Close-up SEM image of re-entrant structures with circular caps and a gap of 20 µm. Scale bar: 5 µm.

The re-entrant structures, including size and surface topology, were analyzed with scanning electron microscopy (SEM) (Figure 2 and Figure 3). Using SEM with an energy-dispersive detector, we verified that the cap materials were SiC and SiO_2_ (please refer to the Supplementary Materials Section for more details). The dimensions, such as the spacing between caps and their heights, were measured with a 3D Measuring Laser Microscope (Olympus OLS5100, Evident Corporation, Tokyo, Japan). The results show that the fabricated circular cap microstructures closely match the intended designs, with only minor deviations. The geometric parameters of these structures are provided in Table 1, which also includes surface free energy values from our previous paper [19]. The gap spacings of 5, 10, and 20 µm were chosen to align with the re-entrant structure dimensions (10 µm stem height and 20 µm cap diameter), balancing stability and separation. These gaps are small enough to maintain pinning and support the Cassie–Baxter state without excessive spacing that could lead to a breakthrough to the Wenzel state, yet large enough to avoid cap overlap, allowing distinct separation between structures. Larger gaps would reduce solid area fraction and pinning points, increasing the likelihood of liquid infiltration between structures, thus compromising superhydrophobicity. This range ensures optimal droplet interactions for studying wettability and evaporation dynamics.

## 3. Results and Discussion

### 3.1. Evaporation Dynamics and Wettability Analysis on SiC and SiO_2_ Flat Surfaces

The investigation aimed to analyze the evaporation dynamics and wettability characteristics of droplets on SiC and SiO_2_ surfaces. Using normalized contact radius, contact angle, and droplet volume as key metrics, we evaluated how each surface interacts with evaporating droplets over time. Wettability, influenced by factors such as surface chemistry and structure, is commonly associated with SFE. Higher SFE correlates with higher wettability (more hydrophilic), while lower SFE indicates greater hydrophobicity [19]. Our study found that the evaporation behavior and droplet volume reduction varied significantly between SiC and SiO_2_, with the SiC surface promoting quicker retraction and volume reduction during evaporation. The detailed discussion is outlined below.

**Figure 4:**
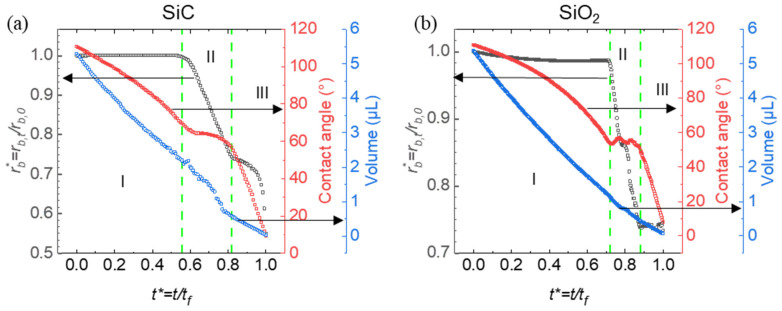
Evaporation dynamics of droplets on flat structures of SiC (**a**) and SiO_2_ (**b**), showing normalized contact radius ($r_{b}^{*}$, black curves), contact angle (red curves), and droplet volume (blue curves) as a function of normalized time $t^{*}$. The green dashed lines indicate key transition points during the evaporation process. The green dashed lines mark the boundaries between three distinct evaporation modes: constant contact line (CCL) (I), constant contact angle (CCA) (II), and mixed mode (III).

The results reveal distinct evaporation phases and differences in droplet volume reduction, with SiC demonstrating a faster shift through evaporation modes compared to SiO_2_. This suggests that surface hydrophobicity and structure play a vital role in determining evaporation rates and wettability characteristics on re-entrant structures (Figure 4).

This graph presents the evaporation dynamics of droplets on with SiC (left) and SiO_2_ (right) flat surfaces (surfaces without any micro-structuring or texturing) with normalized contact radius $r_{b}^{*}$, contact angle, and droplet volume plotted over normalized time. Three distinct evaporation modes are observed: constant contact line (CCL), constant contact angle (CCA), and mixed mode. In the CCL mode, the contact line remains fixed while the contact angle decreases, indicating that evaporation primarily reduces the droplet height. The CCA mode, in contrast, maintains a steady contact angle while the contact line retracts, suggesting that the droplet footprint shrinks as evaporation proceeds. The mixed mode involves variability in both the contact line and contact angle, indicating a more complex evaporation influenced by the surface structure and wettability. Green dashed lines mark transitions between these phases, highlighting distinct evaporation behaviors on SiC and SiO_2_.

The shorter CCL phase and extended CCA phase on SiC compared to SiO_2_ can be attributed to SiC’s higher hydrophobicity on re-entrant structures. Due to this hydrophobicity, droplets on SiC likely exist in a Cassie–Baxter state, resting atop the microstructures with minimal surface contact. This configuration facilitates rapid retraction of the contact line, resulting in a quicker transition out of the CCL phase. Quantitatively, the CCL phase on SiC accounts for approximately 55% of the total droplet lifetime, compared to 68% on SiO_2_, indicating a 24% increase in CCL duration on SiO_2_. This percentage increase, defined as the relative difference in CCL duration between SiO_2_ and SiC, reflects the stronger pinning and adhesion forces on the more hydrophilic SiO_2_ surface, which prolongs the CCL phase. However, once the droplet transitions to the CCA phase, the contact angle is less stable on the less hydrophobic SiO_2_, resulting in a shorter duration of 14% for the CCA phase compared to 22% on SiC. This 36% decrease in CCA phase duration reflects the reduced stability of the contact angle on SiO_2_, causing a quicker transition to the mixed mode. In contrast, on SiC, the droplet maintains a steady contact angle as the footprint reduces, with SiC’s hydrophobic surface supporting the CCA mode over an extended period before shifting to the final mixed mode.

On SiO_2_, the more extended CCL phase and shorter CCA phase can likely be attributed to its relatively lower hydrophobicity. The droplet on SiO_2_ may exist in a more Wenzel-like state, where it partially fills the microstructures, increasing the contact area and adhesion to the surface. This adhesion resists retraction of the contact line, thus prolonging the CCL phase duration. These observations underscore how surface hydrophobicity and microstructuring affect the transitions between evaporation modes on SiC and SiO_2_ surfaces, impacting droplet retention, contact dynamics, and overall evaporation rates.

Figure 4 also illustrates the volume reduction in droplets on SiC and SiO_2_ surfaces as they transition through three evaporation modes: constant contact line (Mode I), constant contact angle (Mode II), and mixed mode (Mode III). In Mode I, volume decreases mainly due to a reduction in contact angle, while in Mode II, volume loss is driven by a shrinking contact radius. Mode III features simultaneous changes in both contact angle and radius. The slight differences in the volume curves between SiC and SiO_2_ suggest that surface material properties influence evaporation dynamics, impacting how the droplet volume diminishes across these modes.

### 3.2. Evaporation Dynamics and Wettability Analysis on SiC and SiO_2_ Re-Entrant Structures

This part investigates the evaporation characteristics and wettability of droplets on re-entrant structures with circular caps made of SiC and SiO_2_. By systematically varying the pillar spacing (5 µm, 10 µm, and 20 µm), we aimed to understand how material composition, microstructural geometry, and solid area fraction influence droplet behavior during evaporation. Normalized contact radius, contact angle, and droplet volume were recorded over time to provide quantitative insights into droplet–surface interactions on these microstructured substrates. Wettability, closely linked to SFE and solid area fraction, shows that surfaces with higher free energy and larger solid area fractions tend to be more hydrophilic, while lower free energy and reduced solid area fractions promote increased hydrophobicity.

**Figure 5:**
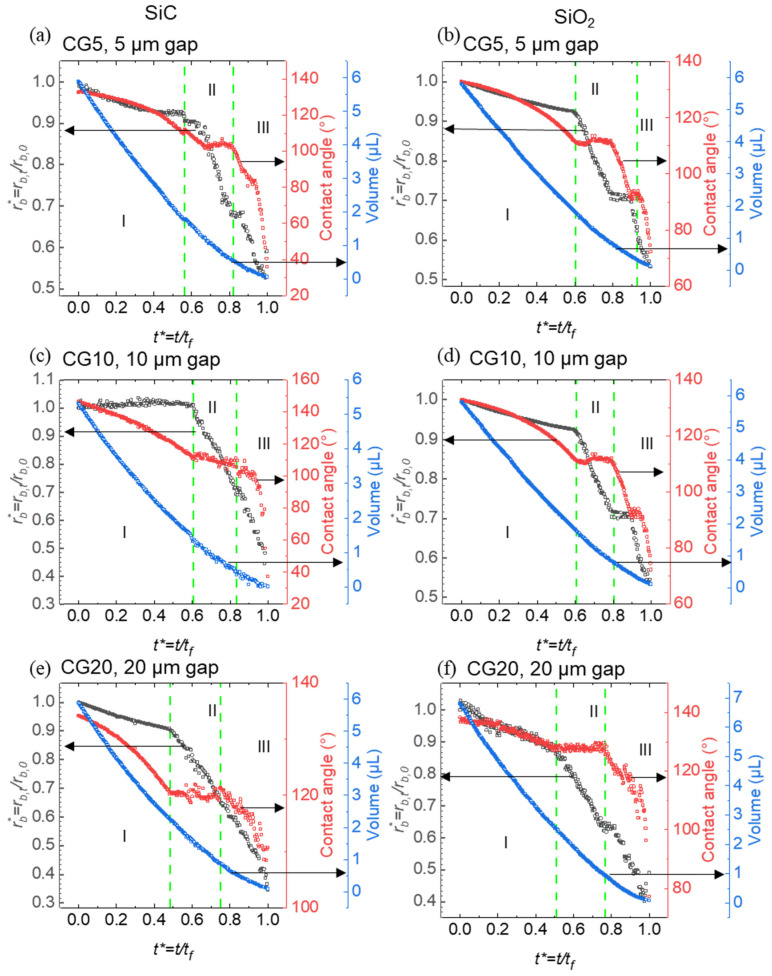
Droplet evaporation dynamics on re-entrant structures with circular caps, showing normalized contact radius ($r_{b}^{*}$, black curves), contact angle (red curves), and droplet volume (blue curves) as a function of normalized time $t^{*}$. (**a**) SiC re-entrant structures with a 5 µm gap between pillars; (**b**) SiO_2_ re-entrant structures with a 5 µm gap between pillars; (**c**) SiC re-entrant structures with a 10 µm gap between pillars; (**d**) SiO_2_ re-entrant structures with a 10 µm gap between pillars; (**e**) SiC re-entrant structures with a 20 µm gap between pillars; (**f**) SiO_2_ re-entrant structures with a 20 µm gap between pillars. The green dashed lines indicate key transitions between evaporation modes, highlighting differences in contact angle, volume, and contact radius across varying materials and pillar spacings.

The results indicate that both the material composition and the pillar spacing significantly impact the progression of evaporation modes and the rate of droplet volume reduction. SiC surfaces generally demonstrated accelerated transitions between evaporation phases, likely due to their hydrophobic response to the structured caps, whereas SiO_2_ surfaces displayed a prolonged constant contact line phase, suggesting a more stable droplet contact area. Additionally, narrower pillar gaps contributed to droplet stability within certain evaporation phases. These findings underscore the importance of material selection and structural design parameters in governing evaporation dynamics on micro-engineered surfaces (Figure 5).

The graph illustrates the evaporation behavior of droplets on SiC and SiO_2_ re-entrant structures with circular caps, across pillar spacings of 5 µm, 10 µm, and 20 µm. Material composition plays a key role in evaporation dynamics, with SiC structures generally showing slightly faster transitions through evaporation modes compared to SiO_2_ structures. This difference is likely due to SiC’s higher hydrophobicity, which allows droplets to retract more readily and shift through evaporation phases. In the CCL phase, SiC with a 5 µm gap (CG5) shows a duration of 55% of the total droplet lifetime, while SiO_2_ CG5 has a slightly extended duration of 59%, indicating a 7% increase in CCL duration on SiO_2_ CG5 compared to SiC CG5. As the gap widens to 20 µm (CG20), the CCL duration on SiC decreases to 49%, while on SiO_2_ CG20, it remains 51%, reflecting a 4% increase in CCL duration on SiO_2_ CG20 compared to SiC CG20.

In the CCA phase, a similar trend is observed. For CG5, the CCA phase accounts for 22% on SiC and 17% on SiO_2_, resulting in a 23% decrease in CCA duration on SiO_2_ CG5 compared to SiC CG5. As the gap widens to 20 µm (CG20), the CCA phase duration stabilizes at 22% on SiC, but increases to 21% on SiO_2_, highlighting the variability in CCA durations as the solid area fraction changes. These differences in CCL and CCA phase durations reflect the impact of hydrophobicity and solid area fraction on pinning effects and contact line stability during evaporation.

A significant factor influencing these evaporation patterns is the solid area fraction of the re-entrant structures, which varies with pillar spacing. As the gap between pillars increases from 5 µm to 20 µm, the solid area fraction decreases, reducing the area available for droplet contact and enhancing hydrophobic effects due to the diminished liquid–solid interaction. For structures with a 5 µm gap, the higher solid area fraction promotes greater droplet adhesion, stabilizing the droplet footprint and slowing contact line retraction. In contrast, structures with a 20 µm gap (CG20) show a reduction in contact area, which facilitates more rapid contact line retraction and quicker transitions through evaporation phases.

This trend emphasizes the interplay between material properties and structural geometry in determining droplet evaporation dynamics. On SiC surfaces, the decreased solid area fraction in wider pillar spacings (10 µm and 20 µm) intensifies hydrophobic behavior, supporting quicker transitions through the CCL and CCA phases. A similar pattern is observed on SiO_2_, though the overall evaporation rate remains slower due to the material’s inherent wettability. Thus, both material hydrophobicity and structural solid area fraction critically influence the progression of evaporation modes, with lower solid area fractions amplifying hydrophobic responses and expediting evaporation dynamics on both SiC and SiO_2_ surfaces.

The comparison between re-entrant structures and other superhydrophobic designs, such as micropillars, underscores the unique advantages of re-entrant geometries in modulating evaporation dynamics. Both this study and the work by McHale et al. (2005) observed prolonged CCL phases, but the nature of the transitions differed markedly [27]. On micropillar surfaces, the prolonged CCL phase was followed by a sharp and rapid collapse into the Wenzel state, as the uniform geometry and less stable hydrophobicity of the micropillars failed to sustain the Cassie–Baxter state under evaporation conditions. This abrupt transition destabilized the evaporation process, leading to irregular shifts between evaporation modes. In contrast, the transitions observed in this study on re-entrant microstructures were smoother and more controlled. The overhanging geometry of re-entrant structures provided enhanced stability to the Cassie–Baxter state by effectively trapping air pockets and reducing liquid infiltration. This stability supported a gradual shift to the CCA mode, reflecting more robust and consistent hydrophobicity. These differences highlight the superior capability of re-entrant designs to maintain hydrophobicity and ensure efficient, controlled evaporation dynamics, setting them apart from conventional micropillar surfaces.

The insights gained from this study on droplet evaporation dynamics and wettability of re-entrant structures have significant implications for various applications. In microfluidics, the ability of re-entrant structures to maintain a Cassie–Baxter state and reduce contact line pinning can enhance the transport efficiency of droplets and prevent contamination by minimizing liquid–solid contact. For thermal management, the observed faster evaporation rates on SiC re-entrant structures suggest their potential for heat dissipation applications, where efficient vaporization of cooling fluids is critical. Similarly, in anti-icing technologies, the strong resistance to wetting provided by these structures can delay ice nucleation and reduce adhesion, preventing ice accumulation on surfaces in cold environments.

### 3.3. Wetting of Re-Entrant Structures

The evaporation dynamics of sessile droplets are governed not only by surface properties and microstructure but also by environmental conditions, particularly temperature and humidity. In this study, experimental conditions were monitored using a temperature–humidity logger and maintained at an average temperature of 26 °C and an average relative humidity of 36%. These conditions reflect typical ambient laboratory settings and were chosen to minimize variability, rather than employing forced elevated temperatures or humidity to accelerate evaporation artificially.

It is well established that environmental parameters significantly influence droplet evaporation dynamics. Higher temperatures increase the saturation vapor pressure, accelerating evaporation and potentially altering heat transfer dynamics at the liquid–solid interface. Conversely, elevated humidity reduces the vapor pressure gradient between the droplet and surrounding air, slowing the evaporation process, particularly in the later stages. On re-entrant surfaces, these factors may also affect transitions between Cassie–Baxter and Wenzel states, influencing contact line dynamics and evaporation modes.

By maintaining consistent environmental conditions, this study isolates the effects of surface properties and microstructure on evaporation dynamics. Future research could explore the impact of varying temperature and humidity on these processes to further expand the understanding of evaporation behavior in diverse environments.

The wettability of re-entrant structures depends not only on surface tension but also significantly on liquid viscosity, which plays a key role in evaporation control. Higher viscosity fluids spread at a slower rate across surfaces, a factor that supports the Cassie–Baxter state by minimizing the likelihood of the liquid penetrating into re-entrant features. This stability impacts evaporation dynamics by maintaining high contact angles that help regulate the evaporation process. Our findings highlight the importance of assessing both surface tension and viscosity when studying how re-entrant structures influence wetting and evaporation behaviors.

To further investigate these dynamics, we calculated the Bond number (Bo) and capillary number (Ca) to gauge the balance between surface tension, gravitational, and viscous forces.

The Bond number is given by $Bo=\frac{∆\rho gr^{2}}{\gamma }$ (where $∆\rho =\rho_{w}-\rho_{a}\approx \rho_{w}$, given that $\rho_{a}$ is negligible compared to $\rho_{w}$, $\rho_{w}$ and $\rho_{a}$ are densities of water and air, g is gravitational acceleration, r is the droplet radius, and γ is the surface tension of the liquid) [31]. With values well below 1, our results suggest that surface tension strongly prevails over gravitational forces, a common characteristic in micro-scale systems. This dominance of surface tension helps maintain the Cassie–Baxter state by sustaining air pockets in the re-entrant structures, contributing to stable evaporation dynamics.

The capillary number is defined as $Ca=\frac{\mu V}{\gamma }$, where μ is the dynamic viscosity of the liquid, *V* is the characteristic velocity of the liquid spreading over the surface, and γ is the surface tension. The capillary number was also low in our experiments (Ca < 0.01). This outcome suggests that surface tension has a stronger influence than viscous forces during wetting, supporting the role of re-entrant geometries in upholding the Cassie–Baxter state and thereby managing evaporation by limiting liquid infiltration.

These dimensionless parameters reveal that surface tension is the predominant force governing the wetting and evaporation behavior in re-entrant structures, underscoring how crucial structural design is to achieve controlled evaporation and maintain superhydrophobicity.

Table 2 shows the solid area fraction for each design (CG5, CG10, and CG20) and their corresponding tilting contact angles for SiC and SiO_2_ materials. As the solid area fraction decreases from 0.5 (CG5) to 0.35 (CG10) and further to 0.19 (CG20), the tilting contact angle also tends to decrease, particularly for CG20. This trend suggests that a lower solid area fraction reduces the surface available for droplet pinning, resulting in a lower tilting contact angle, as seen in CG20, where the tilting contact angles are significantly lower (50° ± 1° for SiC and 63° ± 1° for SiO_2_).

The lower solid area fraction in CG20 facilitates easier droplet movement, reducing pinning forces and enabling the contact line to shift more readily during evaporation. In contrast, higher solid area fractions in CG5 and CG10 (>80° tilting contact angles) promote stronger droplet pinning, likely supporting a constant contact line (CCL) evaporation mode. These results align with our evaporation findings, where CG5 and CG10 designs with higher solid area fractions showed prolonged CCL phases and more stable contact lines. Meanwhile, CG20, with its lower solid area fraction, demonstrated faster transitions between evaporation modes, supporting the conclusion that lower solid area fractions enhance hydrophobic effects and expedite the evaporation process.

The D^2^ and D^3^ laws, which describe droplet evaporation rates based on diameter and volume reduction, apply primarily to smooth, non-textured surfaces with stable evaporation conditions [32]. On textured surfaces, such as those with micropillars or re-entrant structures, evaporation dynamics differ significantly due to effects like contact line pinning, transitions between wetting states (e.g., Cassie–Baxter to Wenzel), and mode shifts (e.g., constant contact radius or angle). Consequently, modified or advanced models are often needed to account for the complex behavior observed on microstructured surfaces.

A theoretical analysis by McHale et al. provides a foundational understanding of how the surface texture and initial contact angle are influenced by re-entrant structures and their material composition [27]. Re-entrant surfaces trap air beneath droplets, often promoting a Cassie–Baxter state where the droplet rests on a composite surface of solid and air pockets. This state is characterized by a high contact angle, following the Cassie–Baxter equation: $\cos\theta_{CB}=\varphi_{s}\cos\theta_{s}-(1-\varphi_{s})$, where $\theta_{CB}$ is the Cassie–Baxter contact angle of the droplet on the textured surface, $\theta_{s}$ is the solid area fraction in contact with the droplet, and $\varphi_{s}$ represents the solid area fraction. This re-entrant geometry effectively reduces the solid–liquid contact area, supporting a superhydrophobic state and high contact angles.

In our study, silicon carbide (SiC) and silicon dioxide (SiO_2_) form the basis for re-entrant structures, where SFE becomes critical. With its lower SFE, SiC stabilizes the Cassie–Baxter state more effectively than SiO_2_. This stability enhances the CCL mode by reinforcing contact line pinning, whereas the higher SFE of SiO_2_ promotes wettability, favoring a shift to the Wenzel state where liquid spreads more across the surface. Relating SFE to these states explains observed differences in evaporation on SiC and SiO_2_, especially in stabilizing the Cassie–Baxter state and maintaining high initial contact angles.

## 4. Conclusions

In conclusion, our study provides a comprehensive view of how material properties, structural design, and solid area fraction collectively influence the wettability and evaporation dynamics of re-entrant structures. By adjusting the gap between microstructural pillars, we demonstrated that a lower solid area fraction enhances hydrophobicity, helping maintain the Cassie–Baxter state and reducing droplet adhesion. Our analysis of SiO_2_ and SiC surfaces, each capped with circular structures across different spacings (5 µm, 10 µm, and 20 µm), reveals that SiC’s higher hydrophobicity encourages faster transitions through evaporation modes, promoting quicker droplet volume reduction compared to SiO_2_. This finding highlights that the inherent hydrophobicity of SiC, combined with optimized solid area fractions, supports efficient evaporation control, as droplets on SiC tend to undergo more rapid retraction and shifts in evaporation phases than those on SiO_2_.

While this study focuses specifically on SiC and SiO_2_ materials due to their distinct wettability and relevance to microfluidic and thermal management applications, future research could explore the impact of alternative surface chemistries. Investigating materials with varying surface energies or incorporating simulations to model droplet dynamics on other chemistries could provide deeper insights into evaporation control across a broader range of applications.

The evaporation dynamics observed illustrate three distinct phases: constant contact line (CCL), constant contact angle (CCA), and mixed mode, each influenced by both material and structural characteristics. On SiC surfaces, the higher hydrophobicity and reduced solid area fraction (seen in 10 µm and 20 µm pillar spacings) facilitate shorter CCL and prolonged CCA phases. This configuration allows for rapid retraction of the contact line and controlled evaporation rates, maintaining the Cassie–Baxter state and limiting liquid–solid interactions. In contrast, SiO_2_’s lower hydrophobicity and increased solid area fraction (especially at 5 µm spacing) result in more stable contact line retention, leading to an extended CCL phase. This behavior suggests that SiO_2_ may promote a more Wenzel-like state, which increases adhesion and prolongs the time droplets remain on the surface.

Our findings underscore the critical role of both material selection and structural design parameters in modulating evaporation dynamics. The decreased solid area fraction in wider pillar spacings (10 µm and 20 µm) amplifies hydrophobic behavior, accelerating evaporation transitions and enabling faster droplet volume reduction, particularly on SiC surfaces. Additionally, by examining the Bond and capillary numbers, our study confirms that surface tension dominates over gravitational and viscous forces in this system, which maintains the Cassie–Baxter state and helps prevent liquid infiltration into the re-entrant structures for future research on optimizing evaporation control through surface engineering, such as exploring alternative materials, surface treatments, and varying re-entrant geometries. While the present work focuses on circular pillar geometries, future research could explore the effects of alternative geometries, such as square or hexagonal pillars, on evaporation behavior. These shapes may further elucidate the role of contact line pinning and depinning during evaporation. Additionally, investigating surface treatments, such as hydrophobic or hydrophilic coatings, could provide deeper insights into the impact of surface energy on evaporation dynamics. Such studies would complement the current findings and extend their applicability to a wider range of materials and microstructural designs.

Future studies could extend these findings by conducting application-specific experiments. For instance, testing the performance of re-entrant structures in microfluidic devices, cooling systems, or anti-icing environments would provide valuable insights into their practical utility. Such investigations would bridge the gap between fundamental research and real-world applications, further enhancing the impact of these designs.

## Figures and Tables

**Table 1:** Pillar diameter (L), gap size (G), solid area fraction, surface free energy, and total evaporation time ($t_{f}$) for circular re-entrant structures with different designs (CG5, CG10, CG20) using SiC and SiO_2_. Each design varies in gap size (G) while maintaining a constant pillar diameter (L = 20 µm).

Design	Material	L (µm)	G (µm)	Solid Area Fraction	Total Evaporation Time ($t_{f}$) (s)	Surface Free Energy (mJ/m^2^)
CG5	SiC	20	5	0.5	2457 ± 28	3.08 ± 0.02
CG10	SiC	20	10	0.35	2294 ± 43	2.74 ± 0.02
CG20	SiC	20	20	0.19	2412 ± 14	1.01 ± 0.01
CG5	SiO_2_	20	5	0.5	2960 ± 32	2.61 ± 0.02
CG10	SiO_2_	20	10	0.35	2556 ± 62	1.87 ± 0.01
CG20	SiO_2_	20	20	0.19	3313 ± 38	1.47 ± 0.01

**Table 2:** The values of tilting CA of water droplets on the re-entrant structures.

	Design	CG5	CG10	CG20
Material				
Solid area fraction		0.5	0.35	0.19
SiC		>80°	>80°	51° ± 1°
SiO_2_		>80°	>80°	63° ± 1°

## Data Availability

Data are provided within the manuscript.

## Supplementary Materials

The following supporting information can be downloaded at: https://www.mdpi.com/article/10.3390/mi15121507/s1, Figure S1: Energy Dispersive Spectroscopy (EDS) spectrum of the SiO_2_ cap material for re-entrant structures, showing peaks corresponding to the elemental composition, including carbon (C), oxygen (O), and silicon (Si).; Figure S2: Energy Dispersive Spectroscopy (EDS) spectrum of the SiC cap material for re-entrant structures, showing peaks corresponding to the elemental composition, including carbon (C), oxygen (O), and silicon (Si). Table S1: Elemental composition of the SiO_2_ cap material for re-entrant structures; Table S2: Elemental composition of the SiC cap material for re-entrant structures.
