# Supplementary material for: The Role of Re-Entrant Microstructures in Modulating Droplet Evaporation Modes
Source: Micromachines (Basel). 2024 Dec 18;15(12):1507. doi: 10.3390/mi15121507 (PMC11676758; doi:10.3390/mi15121507)
Supplement: Supplementary file 1 [file micromachines-15-01507-s001.zip › micromachines-3343176-supplementary.pdf]

Supplementary Materials

The Role of Re-Entrant Microstructures in Modulating Droplet Evaporation Modes

Hoang Huy Vu, Nam-Trung Nguyen * and Navid Kashaninejad *

Queensland Micro- and Nanotechnology Centre, Nathan Campus, Griffith University, 170 Kessels Road, Brisbane, QLD 4111, Australia
* Correspondence: nam-trung.nguyen@griffith.edu.au (N.-T.N.); n.kashaninejad@griffith.edu.au (N.K.)

Si oxidation

A 6-inch silicon wafer was coated with a Low-Pressure Chemical Vapor Deposition (LPCVD) Silicon dioxide (SiO2) film. SiO2 was deposited using an LPCVD furnace, with silane (SiH4) and oxygen (O2) as reactant gases. This process involves heating the furnace to a specific temperature range, usually between 400°C and 500°C, in a controlled low-pressure environment. Silane and oxygen gases are introduced into the chamber, where they react to form a silicon dioxide layer on the wafer surface. The final film thickness was measured at 1000 nm using a benchtop thin-film analyzer (F40, Filmetrics, Inc., CA, USA).

SiC deposition

A 6-inch silicon wafer was coated with a 3C-silicon carbide (3C-SiC) film using an LPCVD furnace. Silane (SiH_4) and propylene (C3H6) served as the reactant gases for this process. The method involves heating the furnace to a temperature of up to 1000°C in a controlled low-pressure environment. The silane and propylene gases are introduced into the chamber, where they react to form a silicon carbide layer on the substrate. The final film thickness, measured using a benchtop thin-film analyzer (F40, Filmetrics, Inc., CA, USA), was 1000 nm.

SiO2 etching

Silicon dioxide was etched using sulfur hexafluoride (SF_6) through a Reactive Ion Etching (RIE) process in an STS ICP etcher. Forward and bias powers were set at 1000 W and 25 W, respectively, with the pressure maintained at 10 mT. For developing the side profile, specifically the stem of the mushroom structure, isotropic etching with SF_6 was performed. During this step, the forward power was increased to 1400 W, platen power to 140 W, and the pressure was maintained at 55 mT. The final cap thickness was approximately 250 nm, measured using a benchtop thin-film analyzer (F40, Filmetrics, Inc., CA, USA).

SiC etching

Silicon carbide was etched using chlorine (Cl_2) through the Reactive Ion Etching (RIE) process in an STS ICP etcher. Forward and bias powers were set at 1200 W and 75 W, respectively, with a pressure of 10 mT. The resulting cap thickness was approximately 250 nm, measured with a benchtop thin-film analyzer (F40, Filmetrics, Inc., CA, USA).

Si etching

To create the side profile (the stem of the mushroom structure), isotropic etching was conducted using SF_6. The process was performed with a forward power of 1500 W and a platen power of 100 W, with the pressure kept at 55 mT.

SiO2 cap material analysis

SiO2 as a cap material for re-entrant structures was analyzed and confirmed by scanning electron microscopy (SEM) with an energy-dispersive (EDS) detector. The details are shown in Table S1 and Figure S1.

Table S1: The elemental composition of the SiO_2 cap material for re-entrant structures

Element	Line	Atomic %	Weight %	Net Counts	Atomic % Error	Weight % Error
C	K	1.3	0.7	200	0.2	0.1
O	K	44.4	31.5	21 222	0.3	0.2
Si	K	54.3	67.8	171 448	0.2	0.3

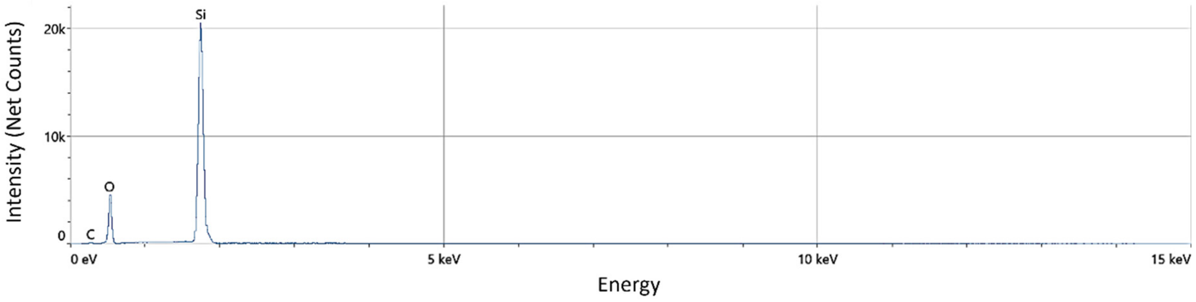

Figure S1: Energy Dispersive Spectroscopy (EDS) spectrum of the SiO_2 cap material for re-entrant structures, showing peaks corresponding to the elemental composition, including carbon (C), oxygen (O), and silicon (Si).

SiC cap material analysis

SiC as a cap material for re-entrant structures was analyzed and confirmed by SEM with an EDS detector. The details are shown in Table S2 and Figure S2.

Table S2: The elemental composition of the SiC cap material for re-entrant structures

Element	Line	Atomic %	Weight %	Net Counts	Atomic % Error	Weight % Error
C	K	53.9	33.3	62 400	0.3	0.2
O	K	0.0	0.0	0	---	---
Si	K	46.1	66.7	465 226	0.2	0.2

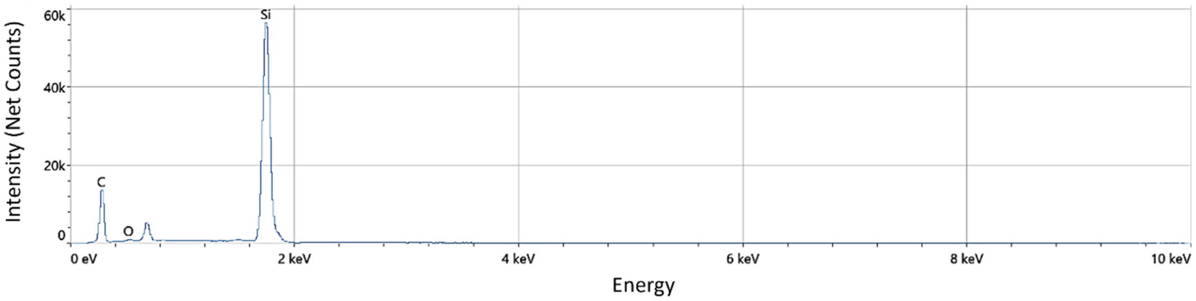

Figure S2: The Energy Dispersive Spectroscopy (EDS) spectrum of the SiC cap material for re-entrant structures, showing peaks corresponding to the elemental composition, including carbon (C), oxygen (O), and silicon (Si).

C4F8 coating and characterization

Re-entrant structures were fabricated and subsequently coated with C_4F_8 using an STS ICP etcher. The process involved a forward power of 875 W and gas flows of 70 sccm for C_4F_8, 1 sccm for SF_6, and 40 sccm for Ar, with the chamber pressure set at 22 mT. The resulting C_4F_8 coating thickness on the re-entrant structures was 150 ± 5 nm, measured with a Dektak surface profilometer.
